# 1415. Allergies to Antimicrobial Agents Among US Females with Uncomplicated Urinary Tract Infection

**DOI:** 10.1093/ofid/ofab466.1607

**Published:** 2021-12-04

**Authors:** Jeffrey Thompson, Alen Marijam, Fanny S Mitrani-Gold, Jonathon Wright, Ashish V Joshi

**Affiliations:** 1 Kantar Health, New York, NY, USA, New York, New York; 2 GlaxoSmithKline plc., Collegeville, PA, USA, Collegeville, Pennsylvania; 3 GlaxoSmithKline plc, Collegeville, PA, USA, Chicago, Illinois

## Abstract

**Background:**

Uncomplicated urinary tract infections (uUTI) are generally treated empirically with antibiotics. However, antibiotic (AB) allergies limit the available oral treatment options for some patients. We assessed the proportion of self-reported AB allergies among US females with uUTI.

**Methods:**

We performed a cross-sectional survey of US females ≥ 18 years of age with a self-reported urinary tract infection (UTI) in the 60 days prior to participation and a prescription of oral AB. Participants were further screened for evidence of a complicated urinary tract infection and, after exclusions, participants with a uUTI completed an online questionnaire about their most recent episode. Participants were from the Northeast (20%), Midwest (44%), South (20%), and West (16%) US. Descriptive self-reported allergy data were stratified into subgroups by whether the participant had recurrent UTI (defined as ≥ 2 uUTIs in the past 6 months or ≥ 3 uUTIs in past 12 months including index UTI), the number of different ABs given for the index episode (1, 2, ≥ 3), and whether the treatment was clinically appropriate according to Infectious Diseases Society of America uUTI guidelines.

**Results:**

Overall, 375 female participants completed the questionnaire. The most commonly prescribed ABs for participants’ most recent uUTI were trimethoprim-sulfamethoxazole (TMP-SMX; 38.7%), ciprofloxacin (22.7%), and nitrofurantoin (18.9%) (Table 1). Most participants received only 1 AB for their uUTI (62.7%) and the majority were classified as having a non-recurrent uUTI (56.5%). No AB allergies were reported for most participants (69.3%); overall, 24.0% reported 1 AB allergy and 6.7% reported ≥ 2. A higher proportion of participants reported ≥ 2 allergies in the recurrent uUTI, ≥ 3 AB, and multiple AB subgroups (Table 2). The most common allergy was to TMP-SMX (15.7%), followed by amoxicillin-clavulanate (8.3%) and ciprofloxacin (5.3%) (Table 2). Similar allergy trends were seen across subgroups, except higher rates of ciprofloxacin allergy were seen in participants given multiple ABs (Table 2).

Table 1. Antibiotics used to treat most recent uUTI

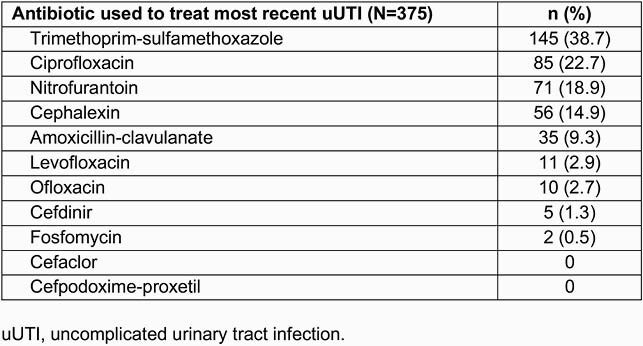

Table 2 . Frequency of antibiotic allergies across cohort subgroups

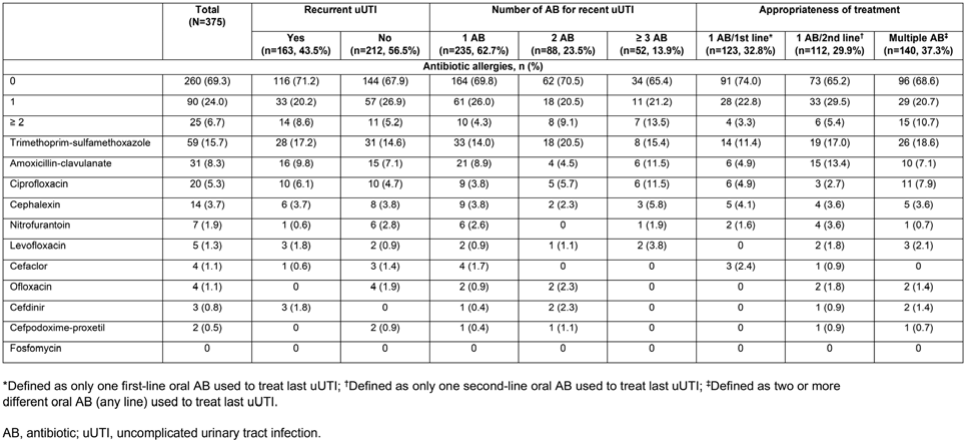

**Conclusion:**

AB allergies were relatively frequent in this uUTI cohort and the most common allergy was to TMP-SMX, which was the most prescribed AB. Allergies to ABs reduce the available treatment options for uUTI in some patients.

**Disclosures:**

**Jeffrey Thompson, PhD**, **Kantar Health** (Employee, Employee of Kantar Health, which received funding from GlaxoSmithKline plc. to conduct this study) **Alen Marijam, MSc**, **GlaxoSmithKline plc.** (Employee, Shareholder) **Fanny S. Mitrani-Gold, MPH**, **GlaxoSmithKline plc.** (Employee, Shareholder) **Jonathon Wright, BSc**, **Kantar Health** (Employee, Employee of Kantar Health, which received funding from GlaxoSmithKline plc. to conduct this study) **Ashish V. Joshi, PhD**, **GlaxoSmithKline plc.** (Employee, Shareholder)

